# Moderate expression of TRF2 in the hematopoietic system increases development of large cell blastic T-cell lymphomas

**DOI:** 10.18632/aging.100015

**Published:** 2009-01-21

**Authors:** Sebastian Begemann, Francesco Galimi, Jan Karlseder

**Affiliations:** ^1^ The Salk Institute for Biological Studies, La Jolla, CA 92037; ^2^ Current address: Biotype AG, Moritzburger Weg 67, 01109 Dresden, Germany; ^3^ Department of Biomedical Sciences / INBB, University of Sassari Medical School, Viale San Pietro 43/C, 07100 Sassari (SS), Italy

**Keywords:** Telomeres, Genome Stability, TRF2, Cancer, Lymphoma

## Abstract

The telomeric repeat binding factor 2 (TRF2) plays a central role in the protection of chromosome ends by inhibiting telomeres from initiating a DNA damage cascade. TRF2 overexpression has been suggested to induce tumor development in the mouse, and TRF2 levels have been found increased in human tumors. Here we tested whether moderate expression of TRF2 in the hematopoietic system leads to cancer development in the mouse. TRF2 and a GFP-TRF2 fusion protein were introduced into hematopoietic precursors, and tested for function. TRF2 overexpressing cells were integrated into the hematopoietic system of C57BL/6J recipient mice, and animals were put on tumor watch. An increase in the development of T-cell lymphomas was observed in secondary recipient animals, however, overexpression of the TRF2 transgene was not detectable anymore in the tumors. The tumors were characterized as large cell blastic T-cell lymphomas and displayed signs of genome instability as evidenced by chromosome fusions. However, the rate of lymphoma development in TRF2-overexpressing animals was low, suggesting the TRF2 does not serve as a dominant oncogene in the system used.

## Introduction

Telomeres are protective caps at chromosome ends that consist of G rich repeats and associated proteins [1,2]. Their major functions are to buffer replication associated shortening, and to protect chromosome ends from being processed as double stranded breaks by the cellular repair machinery. Telomeres can lose their protective function by excessive erosion of the telomeric DNA tracts, as demonstrated in mouse models where the RNA subunit or the catalytic subunit of telomerase have been subjected to targeted deletion [3,4]. When telomeres become critically short they fail to form a protective structure  and are recognized as  DNA damage, leading to cell cycle arrest, repair, or cell death [1,5]. Repair of critically short telomeres, mostly accomplished by the non-homologous end joining  (NHEJ) machinery, results in covalent fusion of chromosome ends [6]. When a cell passes through mitosis with fused chromosomes they break randomly, leading to unequal distribution of DNA to the daughter cells, and hence to genome instability. It has been shown extensively that telomere dysfunction results in unstable chromosomes, and can therefore lead to neoplastic transformation [1,5,7,8].

The core complex of telomere associated proteins is termed shelterin, and consists of TRF1, TRF2, RAP1, TIN2, TPP1 and POT1, and plays a crucial role in telomere protection and the regulation of telomere length homeostasis [2]. Disruption of the complex leads to telomere dysfunction, often without extensive loss of telomeric double stranded DNA. It has been well established that many levels of protection exist and that they interact to inhibit the DNA damage machinery at natural chromosome ends. It is challenging to assign individual roles to the proteins in the complex, since disruption of any component might cause destabilization of shelterin. For example, deletion of TRF1 or TIN2 in mice causes embryonic lethality independent of telomerase dependent telomere length regulation [9,10]. Since TIN2 interacts with both TRF1 and TRF2 [11-13], it is unclear at this point what the exact pathway to lethality is. Similarly, suppression of POT1 led to partial loss of the telomeric 3' single stranded overhang, and a transient detection of telomeres by the DNA damage machinery [14]. The protective effects of POT1 are dependent on its interactions with TPP1 [15], again demonstrating the interdependence of the members of shelterin. POT1 also protect telomeres by preventing activation of the ATR dependent DNA damage response machinery [16]. Inhibition of TRF2 by a dominant negative allele or by targeted deletion leads to extensive loss of the single stranded overhang and to dependent chromosome fusion by NHEJ [6,17-20]. TRF2 also represses the ATM dependent DNA damage response [16], potentially by directly interacting with the kinase [21].

Relatively little is known about the role the shelterin components play in tumorigenesis. TRF1, TRF2 and TIN2 have been found up-regulated occasionally during in gastric carcinomas and during hepatocarcinogenesis [22,23]. Mutations in TIN2 have been demonstrated to lead to abnormally short telomeres, and to be associated with dyskeratosis congenita and ataxia-pancytopenia, diseases associated with an increased cancer disposition [24-26].

Despite the fact that shelterin components interact with proteins involved in many repair processes [2,27-30], no general trend for de-regulation in tumors has been observed. In an effort to study TRF2 overexpression in a mammalian organism mTRF2 has been overexpressed under the K5 promoter in basal and stem cells of the epidermis [31,32]. This led to XPF dependent telomere loss and increased skin cancer levels in the animals [31], a phenotype that was accelerated by telomerase abrogation [32].

TRF2 has also been demonstrated to directly interact with ATM, and overexpression of TRF2 can partially prevent ATM phosphorylation and the activation of the ATM dependent DNA damage response [21,33]. Based on this finding we set out to test whether modest TRF2 overexpression in the murine hematopoietic system leads to a suppression of the DNA damage response and to lymphoma development, as demonstrated for mice lacking ATM [34]. Here we show that TRF2 and GFP-TRF2 can be overexpressed in the hematopoietic system of C57BL/6J mice, and that the transgenic TRF2 localizes to telomeres. Approximately 15% of animals that were secondary recipients of TRF2 overexpressing hematopoietic precursors developed T cell lymphomas. Although lymphoma incident was elevated in this cohort, most mice did not develop cancer during their life-span, suggesting that TRF2 is not a dominant oncogene in this system.

## Results and discussion

### Overexpressed mTRF2 and GFP-mTRF2 localize to telomeres

**Figure 1.**
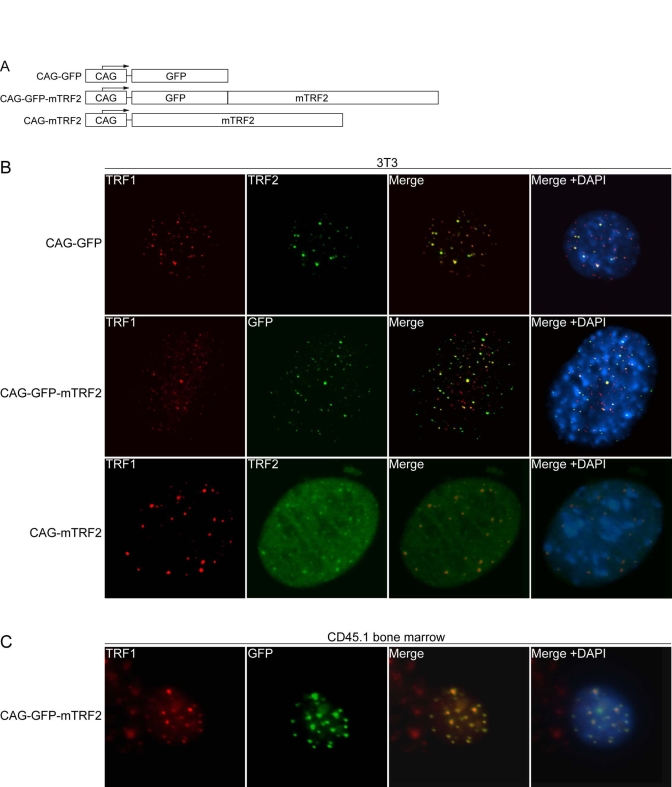
Lentiviral expression of mTRF2 and GFP-mTRF2. **(A)** Schematic of transgene constructs. GFP, mouse TRF2 (mTRF2) and a GFP-mTRF2 fusion were cloned into a lentiviral vector system [35] under the control of a CAG promoter [38]. **(B)** Indirect immunofluorescence of 3T3 cells. 3T3 control cells (top panel), 3T3 cells transfected with GFP-mTRF2 and cells transfected with the mTRF2 construct were stained with antibodies against mTRF1 or mTRF2. GFP was visualized by autofluorescence. DNA has been stained with DAPI, and the merge of the red, green and blue channels has been provided on the right. **(C)** Indirect immunofluorescence of CD45.1 donor bone marrow cells. Cells were infected with GFP-mTRF2 expressing lentiviruses and GFP-mTRF2 was visualized by GFP autofluorescence. TRF1 was detected by a mTRF1 specific antibody, the DNA was counterstained with DAPI And the merge of the three colors is indicated.

Wild type mouse TRF2 (mTRF2), a fusion of GFP and mTRF2, as well as a GFP control were introduced into lentiviral constructs under the control of the CAG promoter (Figure 1A), and the constructs were transfected into murine 3T3 fibroblasts. Indirect immunofluorescence of control 3T3 cells with antibodies against mTRF1 and mTRF2 revealed telomeric co-localization of the two proteins (Figure 1B, upper panel). The GFP-mTRF2 fusion protein also co-localized with endogenous mTRF1, demonstrating that the fusion protein localizes to telomeres (Figure 1B, middle panel). Similarly, overexpressed mTRF2 localized to telomeres (Figure 1B, lower panel), as detected by immunofluorescence with antibodies against mTRF1 and mTRF2. To test whether mTRF2 was also expressed and localizes to telomeres in hematopoietic precursors, high titer lentiviruses were generated, and a liquid culture of CD45.1 donor bone marrow was infected with the lentiviruses, and expression and localization was tested by immuno-fluorescence. Co-localization of mTRF1 and GFP-mTRF2 demonstrated the telomeric localization of the fusion protein (Figure 1C), as well as the wild type TRF2 allele (data not shown). The cell lines expressing the transgenes did not display altered growth rates or cell death (data not shown), suggesting that the expressed TRF2 alleles do not interfere with telomere protection.

### TRF2 constructs integrate and express in bone marrow and spleen of transgenic C57BL/6J mice

**Figure 2.**
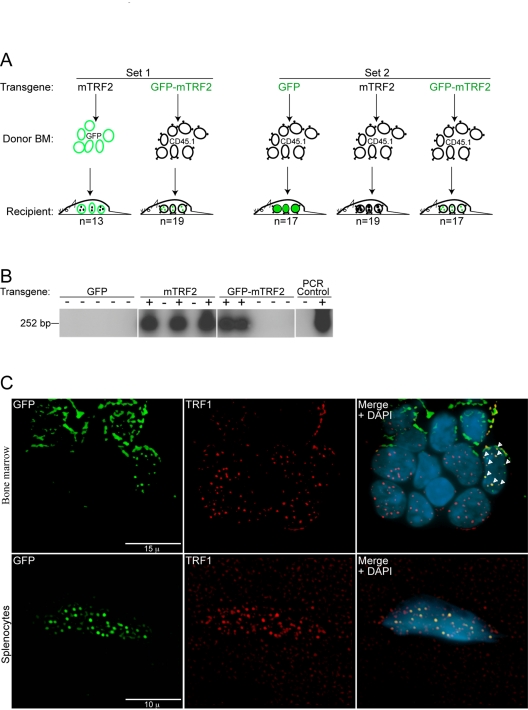
Generation of primary recipient mice by lentiviral transduction of transgenic TRF2 into GFP and CD45.1 donor bone marrow. **(A)** A primary #1 mouse colony (Set 1) was generated by the transduction of GFP donor bone marrow (BM) with the mTRF2 transgene and CD45.1 donor bone marrow with the GFP-mTRF2 transgene. For the primary #2 colony (Set 2) only CD45.1 donor bone marrow was used and transduced with a GFP-control, the mTRF2 or the GFP-mTRF2 transgene. Recipient mice were C57BL/6J. **(B)** Genotyping of primary recipient mice transduced with transgenic GFP-mTRF2 and GFP by nested PCR and subsequent Southern analysis. Examples for Set 1 and Set 2 are displayed. As a negative control (-) genomic DNA from a C57BL/6J mouse was used. As a positive PCR control (+) genomic DNA of GFP-mTRF2 expressing HeLa cells was included. **(C)** Transgenic GFP-mTRF2 is expressed in the hematopoietic system of recipient C57BL/6J mice. Indirect immunofluorescence of bone marrow (top) and spleen (bottom) isolated from a secondary recipient of GFP-mTRF2 expressing bone marrow. GFP-mTRF2 was visualized by the GFP-tag, TRF1 was detected by a mTRF1 specific antibody. Chromatin was counterstained with DAPI. White arrows indicate co-localization of endogenous TRF1 with recombinant GFP-mTRF2.

Donor bone marrow was infected in two independent sets of experiments, where either GFP positive donor cells or CD45.1 expressing donor cells were used for infection. This approach allows identification of the transplanted cells in the recipient bone marrow by FACS analysis later. In the first set GFP positive donor bone marrow was infected with lentiviruses expressing wild type mTRF2 and then re-introduced into the tail veins of 13 lethally irradiated C57BL/6J donor mice. Alternatively, CD45.1 bone marrow was infected with GFP-mTRF2 expressing lentiviruses, and was injected into the tail veins of 19 lethally irradiated C57BL/6J donor mice (Figure 2A, left panel).

In the second set only CD45.1 donor bone marrow was used, and 17 mice were generated that expressed a GFP control, 19 that received cells that expressed wild type mTRF2, and 17 that were transduced with cells expressing the GFP-mTRF2 fusion (Figure 2A, right panel).

After recovery and repopulation of the bone marrow with donor cells, we tested the presence of the transgene in DNA isolated form whole blood of the recipient animals. Using a PCR based approach (Figure 2B) followed by southern analysis 26 mice of the first infection-set tested positive for the presence of the transgene in blood, as well as 15 animals of the second set. Mice transplanted with bone marrow that was infected with GFP control viruses did not give a signal in the PCR-southern analysis. In summary, we generated 41 animals that expressed mTRF2 or mTRF2-GFP transgenes in their bone marrow.

### Transplantation of bone marrow from primary recipients into secondary C57BL/6J recipient mice

To further promote tumor progression in recipient mice, we transplanted bone marrow from primary recipient mice that were successfully transduced with transgenic TRF2. Primary bone marrow from both primary sets was isolated four months post infection and transplanted into lethally irradiated C57BL/6J secondary recipients. A total of five secondary recipient populations were generated: two populations with a total of 26 animals received bone marrow expressing mTRF2, another two populations with a total of 28 animals received bone marrow expressing GFP-mTRF2. As negative control a population of 30 animals received bone marrow transduced with the GFP transgene.

To test for functionality of the TRF2 alleles in the integrated cell populations we isolated bone marrow as well as splenocytes from secondary recipient animals. Immunofluorescent staining demonstrated clear co-localization of GFP-TRF2 with TRF1 in bone marrow cells (Figure 2C, upper panel) and splenocytes (Figure 2C, lower panel), suggesting telomeric localization and functionality. In summary, transgenic mTRF2 integrates into donor cells, which keep expressing the transgene after repopulating the bone marrow of lethally irradiated recipient animals.

### Development of T-cell lymphoma without TRF2 overexpression

**Figure 3.**
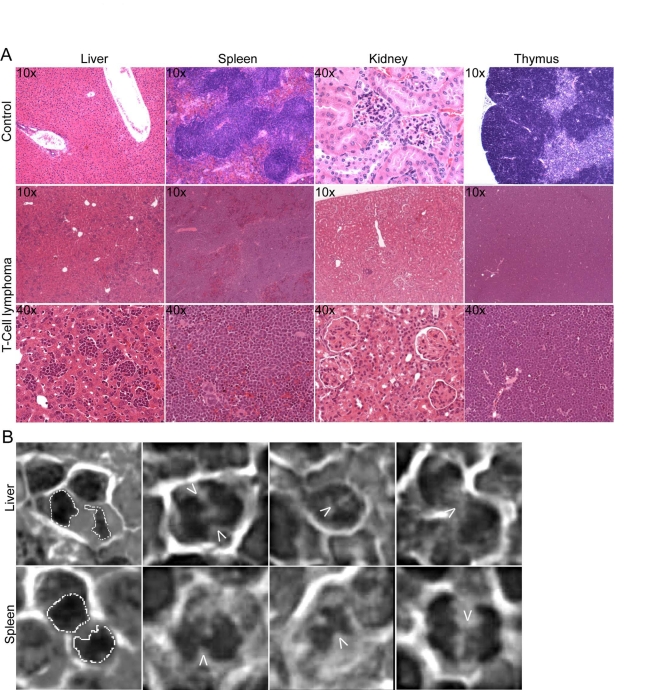
Development of genetically unstable T cell lymphoma in TRF2 overexpressing mice. **(A)** H&E staining of tumor tissue. Tissue samples from a secondary recipient mouse, which developed a blastic T cell lymphoma, involving liver, spleen and thymus. 10x and 40x magnifications are shown. The upper panels show sections of control tissue from a healthy C57BL/6J mouse. **(B)** Anaphase bridges in H&E stained liver and spleen sections from a secondary recipient mouse carrying a CD4/CD8+/+ T cell lymphoma. The arrows point to the chromatin bridges between the separating chromosomes. A dashed line outlines normal anaphases displayed in the images to the very left.

A modest increase in the development of T-cell lymphoma was observed in secondary recipient mice that were transduced with TRF2 expressing cells within 12-month post transplantations, as opposed to GFP control cells. 8 out of 54 mice (14.8%) that tested positive for mTRF2 transgenes succumbed to visible tumors within 52 weeks of transplantation, whereas none of the 30 control mice that carried the GFP transgene displayed visible tumors within the same timeframe. Tissue samples from a secondary recipient mouse suggested development of a large cell blastic T-cell lymphoma (Figure 3A, lower two panels). Spleen, liver and thymus were infiltrated and the organ cells replaced by a diffuse monotonous neoplastic infiltrate composed of cells with large oval nuclei with a delicate chromatin pattern and spare cytoplasm. The cells had a high mitotic index, but also displayed the foci characteristic for apoptosis. The renal glomeruli displayed thickening of the basement membrane and some were hyalinized. The upper panels show control tissue from a healthy C57BL/6J mouse.

A hallmark of tumors and telomere-dysfunction derived tumors is genome instability, resulting from chromosomal breakage fusion cycles. To analyze the T-cell lymphomas for fused chromosomes as indicators of genome instability we screened pathological samples for fused chromosomes, visible as anaphase bridges. Spleen and liver samples readily displayed anaphases where the sets of daughter chromosomes were connected by DNA bridges, suggesting that breakage fusions cycles occur, and the genome in the tumors is unstable  (Figure 3B).

Analysis of the thymomas by flow cytometry with the markers CD4 and CD8 suggested the presence of a donor derived CD4/CD8+/+ T cell lymphoma. However, even when the donor cells were derived from mice that had been transduced with GFP-mTRF2 expressing bone marrow, less than 1% of total thymocytes were positive for GFP (data not shown). These experiments raised the possibility that the observed CD4/CD8+/+ T cell lymphomas originated from the CD45.1 donor population expressing the GFP-mTRF2 transgene, but at the time of analysis most tumor cells did not overexpress mTRF2 anymore, raising the possibility that TRF2 overexpression is a cancer initiating, but not a cancer maintaining event.

**Figure 4.**
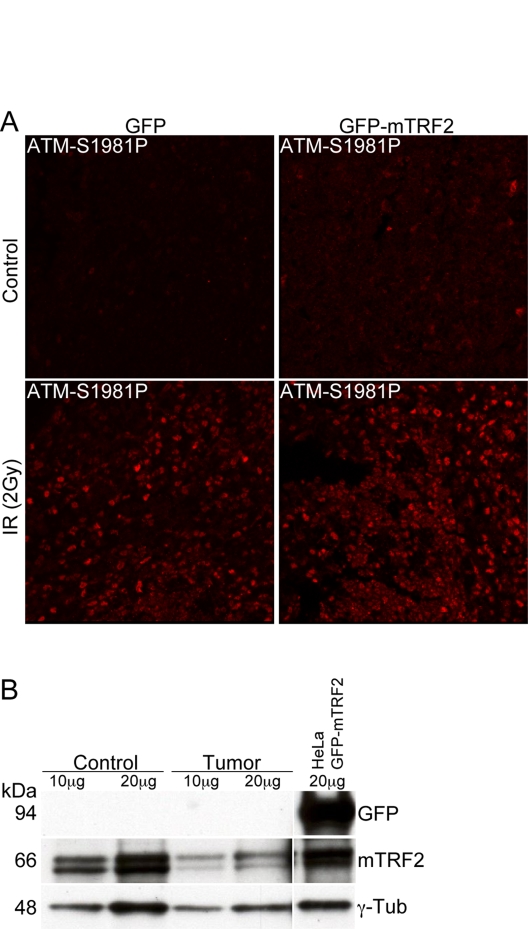
The ATM dependent damage response is not compromised in tumor samples. **(A)** Immunofluorescence of ATM autophosphorylation after ionizing irradiation. Splenocytes were irradiated with 5 Gy and microtome sections were prepared as described in the "Materials and Methods" section.  ATM activation was measured by immunofluorescence with an antibody specific for the phosphorylation event at serine 1981. The left panels represent cells from a control animal, the right panel from an animal expressing the GFP-mTRF2 fusion. The upper panels are before, the lower panels after irradiation. **(B)** Western analysis of spleen from a secondary recipient mouse, which expressed GFP-mTRF2 and died from a CD4/CD8+/+ T cell lymphoma. Protein samples were probed with antibodies against GFP and mTRF2. g-Tubulin was included as a loading control. As a positive control for GFP expression protein extract from HeLa 1.2.11 cells expressing GFP-mTRF2 was loaded.

TRF2 has been proposed to directly interact and suppress ATM activation. The underlying hypothesis of this study was to test whether TRF2 dependent ATM suppression can lead to tumorigenesis. Therefore we tested whether ATM auto-phosphorylation was compromised in the tumors resulting from TRF2 expression in the hematopoietic system. Splenocytes from GFP control mice, as well as cells from enlarged spleens in GFP-mTRF2 expressing mice  were  isolated, cultivated, and subjected to ionizing irradiation. Then ATM activation was tested by immunofluorescence with antibodies specific for the ATM-S1981 autophosphorylation event. No difference in ATM autophosphorylation could be observed between the samples, suggesting that ATM activation is not compromised in tumors resulting from overexpression of TRF2 in hematopoietic precursors (Figure 4A).

Finally we investigated by western analysis whether TRF2 was still overexpressed in the tumors, and we tested GFP-mTRF2 expression in splenocytes isolated from a mouse affected by a T cell lymphoma. No band could be observed in western analysis with an anti-GFP antibody (Figure 4B, upper panel) that readily recognizes the GFP-mTRF2 fusion expressed in HeLa 1.2.11 cells (upper panel, right lane). However, endogenous TRF2 levels, normalized to the g-tubulin loading control, were equal. Our data therefore suggest that mTRF2 is not overexpressed anymore in the lymphomas observed in recipient mice.

In summary, moderate overexpression of TRF2 in hematopoietic precursors in mice leads to an increase of tumor incident in affected animals. Tumors were characterized as CD4/CD8+/+ T cell lymphomas, and they exhibited anaphase bridges, strongly suggesting the possibility of  genome instability.  The modest  increase in cancer formation is contrary to the strong increase in tumor numbers observed upon overexpression of TRF2 in basal and stem cells of the epidermis [31,32], which led to XPF dependent increased skin cancer levels in the animals [31], suggesting a less severe impact of increased TRF2 levels in the hematopietic system than in the epidermis. Furthermore, we observed that the tumors resulting from transduction with TRF2 overexpressing cells do not exhibit TRF2 over-expression in most of their cells, raising the possibility that increased TRF2 expression is a driving event for cancer formation, but not required for tumor maintenance.

## Materials and methods

Constructs and virus production. Lentiviral constructs were generated using standard cloning procedures. The viral backbones p156RRLsinPPTCAG-EGFP-PRE and p156RRLsinPPTmCMV-GFP-PRE [35] were kindly provided by the Verma laboratory.

Lentivirus production. 293T cells were plated on one 15 cm plate and grown in 1x DMEM supplemented with 10% (v/v) FBS, non-essential amino acids (0.1 mM), Penicillin (100 units/ml) and Streptomycin (0.1 mg/ml) and grown to confluence. Cells were trypsinized and split into twelve 15 cm plates coated with poly-L-lysine (Sigma). When cells reached about 70% confluence 95 μg of pVSVG, 68 μg of pREV, 176 μg pMDL, and 270 μg transgene containing lentiviral vector were mixed. Under swirling CaCl_2_ solution was added to a final concentration of 0.25 M. Subsequently an equal volume of 2x BBS solution to the calcium-DNA mixture was added. The mixture was incubated for 10 minutes at room temperature, added drop wise at a volume of 2.25 ml per 15 cm plate and cells were incubated at 3% CO_2_at 37^o^C. The medium was exchanged 12 to 16 hours post transfection and virus-containing media was harvested at 24 h intervals twice, beginning 24 hours after changing the medium. Every sample was immediately filtered through a 0.22 μm cellulose acetate filter and stored at 4^o^C. The collected medium was loaded into ultracentrifuge tubes and spun in a SW28 rotor for 2 hours at 19400 rpm in a L8-80M ultracentrifuge (Beckman). The supernatant was poured off and remaining medium drops were aspirated from the tubes. The pellet was resuspended in 1 ml HBSS, all pellets from two collections were pooled and loaded on top of 1.5 ml of phosphate-buffered 20% (w/v) sucrose in small ultracentrifuge tubes. Tubes were then spun in the SW55 rotor for 2 hours at 21000 rpm in a L8-80M ultracentrifuge (Beckman). Supernatant was removed and pellet resuspended in 200 μl in HBSS. The virus suspension was vortexed for 1 to 2 hours at low speed at room temperature, quick-spun in microcentrifuge for 2 seconds, the supernatant aliquoted in 20 μl aliquots and stored at -80^o^C. Virus titer was determined by the p24 ELISA kit (PerkinElmer) according to the manufacturer.

Mouse strains. B6.SJL-Ptprc^a^Pep3^b^/BoyJ mice (The Jackson Laboratory) were used to isolate bone marrow positive for CD45.1, TgN(beta-act-EGFP) mice [36] (a gift from the Verma lab, The Salk Institute) were used to isolate GFP-positive donor bone marrow. As recipient mice, C57BL/6J (The Jackson Laboratory) were chosen.

Isolation of bone marrow. For the generation of the "Set 1" cohort bone marrow was isolated from 15 male B6.SJL-Ptprc^a^Pep3^b^/BoyJ mice (CD45.1 donor, The Jackson Laboratory) and 5 male TgN(beta-act-EGFP) mice (GFP donor, a gift from the Verma lab, The Salk Institute). For the generation of the "Set 2" population done bone marrow was isolated from 20 male B6.SJL-Ptprc^a^ Pep3^b^/BoyJ animals (CD45.1 donor, The Jackson Laboratory). The mice were sacrificed by cerebral dislocation and femur and tibia were placed into 1x PBS/2 % (v/v) BIT9500 (StemCell Technologies). To isolate the bone marrow, femur and tibia were mortared, the suspension filtered through a Cell Strainer (BD Falcon) and centrifuged for 10 minutes at 700 x g. The pellet was resuspended in 1x PBS and cell numbers were determined by counting a 1:20 dilution of the suspension using a Coulter Counter. Suspensions were diluted to 5x10^7^ cells/ml. To enrich hematopoietic stem cells, cell suspensions were separated using the StemStep^TM^ cell separation system (StemCell Tech-nologies) as directed. The cell numbers of the enriched hematopoietic stem cells were determined and resuspended in Myelocult M5300 (StemCell Tech-nologies).

Infection of bone marrow. Sorted bone marrow cells were diluted to approximately 1.2x10^7^ to 1.4x10^7^cells/ml in Myelocult M5300 medium and 200 ml virus was added to the cells. The suspension was incubated at 37^o^C o/n and then the suspension was washed once with 1x HBSS, centrifuged for 5 minutes at 400 x g, and resuspended in 1x HBSS.

Transplantation of bone marrow. Prior to transplantation recipient C57BL/6J mice were irradiated with 11Gy and subsequently deeply anesthetized. Each mouse received lateral tail vein injections of 100.000 to 200.000 cells diluted in 300 ml 1x HBSS. During the first two weeks post transplantation all mice were maintained on Baytril water (Bayer Health Care). All mice were stored in the Biohazard suite at the Salk Institute's Animal Facility throughout the course of the experiment.

Genotyping of primary and secondary recipient mice. Genomic DNA from blood and tissue samples of C57BL/6J mice as well as HeLa 1.2.11 expressing GFP-mTRF2 cells was isolated using the DNeasy tissue kit (Qiagen). Nested PCR was performed with the outer primer pair (mTRF2 Outer F1: 5'-GCA GAT TGC TGT TGG AGG AGG-3'; WPRE R1: 5'-gcc aca act cct cat aaa gag aca g-3') generating a 626 bp PCR-product, followed by PCR with the inner pair (mTRF2 Inner F1: 5'-ATG TCA GCA TCC AAG CCC AGA G-3'; mTRF2 Inner R1: 5'-CCA GTT TCC TTC CCC GTA TTT G-3') generating a 252 bp PCR-product. Integration of the transgene into the hemato-poietic system of primary recipient mice was verified by nested PCR and the PCR-product was separated on a 1.3% (w/v) Agarose gel. The gel was blotted onto a Hybond-N+ nitrocellulose membrane, (Amersham) following standard Southern analysis procedures, using the mTRF2 cDNA as probe.

Protein isolation. Primary cells were washed with 1x PBS on the plate and trypsinized using 2.5% (v/v) Trypsin/EDTA. Cell numbers were determined with a Coulter Counter. Cells were spun for 5 minutes at 1000 rpm and washed twice in 1x PBS and the cell pellet was resuspended at a dilution of 10000 cells/ml in 4x NuPAGE LDS sample buffer (Invitrogen).

Tissue samples were mashed through a 70 mm Cell Strainer (BD Falcon) with the rubber end of a syringe in the presence of 1x PBS/2% (v/v) FCS. Cell numbers were determined with a Coulter Counter.  The cell suspension was then spun for 5 minutes at 1000 rpm and washed twice in 1x PBS. The cell pellet was resuspended at a dilution of 10000 cells/μl in 4x NuPAGE LDS sample buffer (Invitrogen).

Western blotting. Whole cell extracts of primary cells or protein extracts isolated from tissue samples in 4x NuPAGE LDS sample buffer were separated on 3-8% (w/v) Tris-Acetat gradient gels (Invitrogen) and transferred to nitrocellulose. Blocking and incubation with primary and secondary antibodies was performed in 5% (w/v) milk and 0.1% (v/v) Tween in 1x PBS. Antibodies: rabbit-anti-mTRF2 #6889 (1/1000, Karlseder lab), mouse-anti-g-Tubulin GTU-88 (1/10000, Sigma), mouse-anti-GFP (1/200, Chemicon International). After incubation with secondary antibodies (1/5000, Amersham), all blots were developed using the ECL kit (Amersham).

Immunofluorescence on cultured cells, bone marrow and spleen. Immunofluorescence on cultured cells was per-formed as described  [21,37].  Bone marrow  and  spleen suspension from C57BL/6J mice were attached to microscope slides by loading 200 μl of cell suspension into cytofunnels (Thermo Electron Corporation) and centrifugation in a Shandon Cytospin 4 Cytocentrifuge (Thermo Scientific) for 10 minutes at 800 rpm. Primary antibodies: rabbit-anti-mTRF1 #6888 (1/500, Karlseder lab), rabbit-anti-mTRF2 #6889 (1/500, Karlseder lab), mouse-anti-TRF2 (1/500, upstate biotechnology). Secondary antibodies: donkey-anti-rabbit-FITC (1/200, Jackson), donkey-anti-mouse-FITC (1/200, Jackson), donkey-anti-rabbit-TRITC (1/200, Jackson). Pictures were taken on an Axioplan2 Zeiss microscope with a Hamamatsu digital camera supported by OpenLab software.

Immunofluorescence on microtome sections. Tissue sections of mice were isolated and fixed in phosphate-buffered 4% (v/v) formaldehyde and transferred to phosphate-buffered 30% (w/v) sucrose after one day. Sections were blocked with 3% (v/v) FCS in TBS with 0.25% (v/v) Triton-X 100 for 1 hour and incubated o/n at 4^o^C. Primary antibody: rabbit-anti-ATM pS1981 (1/500, Rockland). Then the sections were rinsed in 3% (v/v) FCS in TBS with 0.25% (v/v) Triton-X 100. The sections were incubated with the second antibody in 3% (v/v) FCS in TBS with 0.25% (v/v) Triton-X 100 for 1 to 2 hours, followed by three washing steps in TBS. Secondary antibody: goat-anti-rabbit-TRITC (1/200, Jackson). To stain DNA the sections were incubated in a 1/30000 dilution of 4', 6'-diamidino-2-phenylindole (DAPI) in TBS for 5 minutes. Sections were mounted on coverslip using Dabco/PVA, dried over night at 4 ^o^C in the dark, and sealed with nail polish. Pictures were taken on a Leica TCS SP2 AOBS and analyzed by LCS Lite software.

Flow cytometry . Aliquots of bonemarrow cells and thymocytes were stained with anti-mouse CD45.1 antibody (A20)conjugated to R-Phycoerythrin (R-PE) to detect CD45.1 donor bone marrow. If mice were transplanted with GFP-donor bone marrow, the presence of the GFP signal was used to evaluate the presence of donor bone marrow. Lineage analysis was performed by double staining using anti-mouseCD45.1 R-PE antibody with each of the following antibodies: CD4 (RM4-5.B) and CD8 (53-6.7). Primary antibodies were purchased from BD Biosciences, the secondary antibody was purchased from Molecular Probes. Flow cytometric analysis was performed on a LSR I 3-laser 6-color analytical flow cytometer (Becton-Dickinson) and data were analyzed using the CellQuest software (Becton-Dickinson).

Pathology. Tissue samples of mice were fixed in 4% (v/v) p-formaldehyde, transferred to phosphate-buffered 30% (w/v) sucrose and stored at 4^o^C in the dark. Pathological studies were carried out at the Department of Pathology, UC Davis.
